# Rice Growth Estimation and Yield Prediction by Combining the DSSAT Model and Remote Sensing Data Using the Monte Carlo Markov Chain Technique

**DOI:** 10.3390/plants14081206

**Published:** 2025-04-14

**Authors:** Yingbo Chen, Siyu Wang, Zhankui Xue, Jijie Hu, Shaojie Chen, Zunfu Lv

**Affiliations:** 1 Zhejiang A&F University, Lin’an, Hangzhou 311300, China; 20090050@zafu.edu.cn (Y.C.); 2023601022029@stu.zafu.edu.cn (S.W.); 2 Jinhua Agricultural Technology Promotion and Seed Management Center, Jinhua 321000, China; 18368021906@163.com; 3 Ningbo Agricultural Technology Promotion Station, Ningbo 315800, China; 17716069330@163.com (J.H.); 18389963101@163.com (S.C.)

**Keywords:** crop environment resource synthesis for rice model, remote sensing data, monte carlo markov chain technique, rice

## Abstract

The integration of crop models and remote sensing data has become a useful method for monitoring crop growth status and crop yield based on data assimilation. The objective of this study was to use leaf area index (LAI) values and plant nitrogen accumulation (PNA) values generated from spectral indices to calibrate the Decision Support System for Agrotechnology Transfer (DSSAT) model using the Monte Carlo Markov Chain (MCMC) technique. The initial management parameters, including sowing date, sowing rate, and nitrogen rate, are recalibrated based on the relationship between the remote sensing state variables and the simulated state variables. This integrated technique was tested on independent datasets acquired from three rice field tests at the experimental site in Deqing, China. The results showed that the data assimilation method achieved the most accurate LAI (R^2^ = 0.939 and RMSE = 0.74) and PNA (R^2^ = 0.926 and RMSE = 7.3 kg/ha) estimations compared with the spectral index method. Average differences (RE, %) between the inverted initialized parameters and the original input parameters for sowing date, seeding rate, and nitrogen amount were 1.33%, 4.75%, and 8.16%, respectively. The estimated yield was in good agreement with the measured yield (R^2^ = 0.79 and RMSE = 661 kg/ha). The average root mean square deviation (RMSD) for the simulated values of yield was 745 kg/ha. Yield uncertainty from data assimilation between crop models and remote sensing was quantified. This study found that data assimilation of crop models and remote sensing data using the MCMC technique could improve the estimation of rice leaf area index (LAI), plant nitrogen accumulation (PNA), and yield. Data assimilation using the MCMC technique improves the prediction of LAI, PNA, and yield by solving the saturation effect of the normalized difference vegetation index (NDVI). This method proposed in this study can provide precise decision-making support for field management and anticipate regional yield fluctuations in advance.

## 1. Introduction

The integration of crop models and remote sensing data has become a useful method for monitoring crop growth status and crop yield based on data assimilation over extensive regions. In this process, weather models and weather stations play a foundational supporting role. Weather models provide spatiotemporally continuous predictions of key climatic variables (e.g., precipitation, temperature) at regional scales, while widely distributed weather stations calibrate and validate model outputs through field observations. This integration ensures the accuracy of input data, thereby significantly enhancing the predictive precision of crop models and strengthening their practical applicability [1]. The GreenSeeker™ optical sensor (GS) is a highly effective tool for site-specific nitrogen fertilizer management tailored to specific needs. Vegetation indices, especially the normalized difference vegetation index (NDVI), which is calculated using reflectance in the red and near-infrared bands, are among the most commonly utilized indicators [2]. The NDVI can estimate crop leaf area index (LAI), plant nitrogen accumulation (PNA), nitrogen (N) requirement, and grain yield and improve the N use efficiency. However, the NDVI is prone to saturation at moderate to high LAI values or PNA values [3,4]. The saturation effect of the NDVI was mainly due to the canopy closure—the differences in penetration into the canopy between visible light (R) and near-infrared (NIR). Since the total absorption by a canopy in the red range is already between 90% and 95%, further increases in the green leaf area index (gLAI) do not result in any additional changes in the absorption and reflectance [5] or the normalization effect embedded in the calculation formula of this index [2]. In addition, former studies were principally based on the relationship between vegetation indices and yield to predict yield. However, using remote sensing (RS) data alone is not enough to explain the fundamental principles of crop growth and development processes and connect them to crop yield [6]. Moreover, it also gives poor annual performance in the spatial extension because of environmental, soil, and management changes [7].

Crop growth models are extensively utilized for assessing crop growth status and predicting yields [8]. They capture the interactions among genetic potential, environmental factors, and management practices by simulating the dynamic growth patterns of crops [6,9]. On the field scale, each farmer in China has only a few fields. Different sowing times, sowing rates, and fertilizer amounts were used in different fields, which will affect the application and popularization of crop models. Due to the saturation effect, the crop models could continuously estimate crop LAIs and PNAs and make up for the shortage of spectral monitoring. Therefore, the integration of crop models and remote sensing data has become a useful method for monitoring crop growth status (leaf area index, LAI, and accumulated nitrogen uptake, ANU) and crop yield.

Many studies have been carried out using the data assimilation of crop models and remote sensing data using a calibration method, including the Simplex Search Algorithm [10], Maximum Likelihood Solution [11], Very Fast Annealing Algorithm [12], Ensemble Square Root Filter [13,14], Particle Swarm Optimization Algorithm [6,15], and Ensemble Kalman Filter (EnKF) [16,17]. The calibration method was utilized to reduce the discrepancies between the remote sensing data and the simulated crop model data by employing an optimization algorithm. This process aims to enhance the model’s accuracy in reflecting observed conditions [18]. However, the above methods cannot quantify the uncertainty of data assimilation between crop models and remote sensing. The MCMC method is built on the Bayesian theoretical framework, constructing a balanced distribution for the Markov chain and sampling from it. By continuously updating the sample information, the chain can thoroughly explore the parameter space and ultimately converge to areas of high probability density [19]. Compared with the above methods, the MCMC method not only could find the optimal combination of initial parameters but also quantify the range of initial parameters.

The main objective of this study is (1) to improve the estimation of rice leaf area index (LAI), plant nitrogen accumulation (PNA), and yield by combining crop models and remote sensing data using the MCMC technique; (2) to improve the prediction of LAI and PNA by solving the saturation effect of the NDVI; and (3) to quantify the uncertainty of data assimilation between crop models and remote sensing.

## 2. Result

### 2.1. Spectral Index for LAI and VNA Estimation

**Figure 1:**
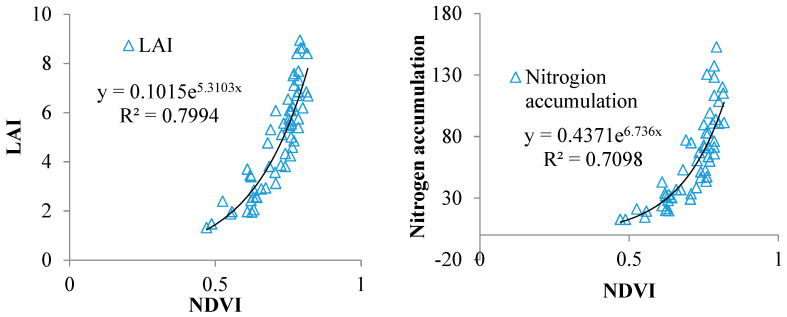
The statistical model between the NDVI and the LAI and PNA.

The data from experiments 1 and 2 were used to build the relationship between the NDVI and the LAI or PNA. The results showed that there was an exponential regression relationship between the NDVI and the LAI or PNA (Figure 1). The NDVI indices could be used to estimate the PNA and LAI in rice.

**Figure 2:**
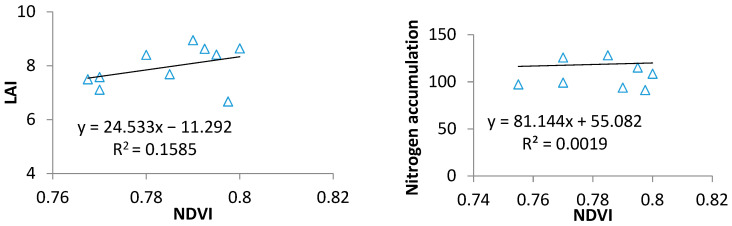
The statistical model between the NDVI and the LAI and PNA when the NDVI was higher than 0.74.

The GreenSeeker NDVI showed a significant correlation with LAI and PNA (R^2^ = 0.79), but saturation occurred when the LAI exceeded 7, becoming nearly constant at values above this threshold (Figure 2). Consequently, the LAI was often underestimated when it surpassed 7. Similarly, across various growth stages and site years, the GreenSeeker NDVI demonstrated a significant correlation with PNA (R^2^ = 0.71), though it reached saturation when the PNA hit 7.0 and remained almost unchanged for PNA values above 90 (Figure 2).

### 2.2. The Probability Distribution of Inverted Initial Parameters

**Figure 3:**
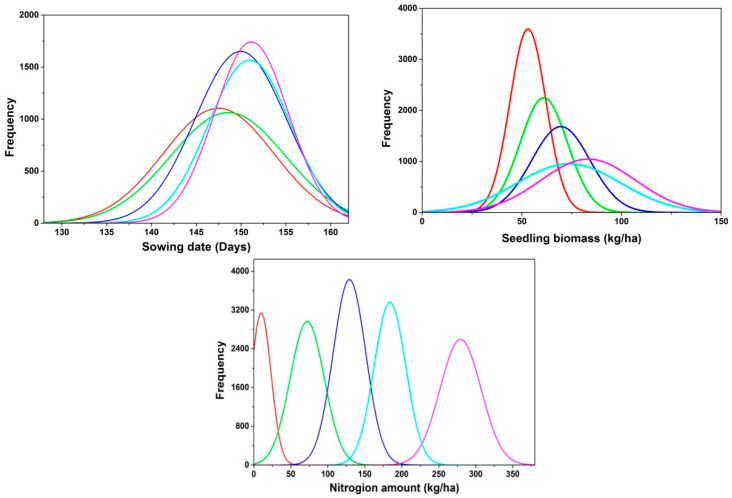
The probability distribution of inverted initial parameters using the MCMC method. Red line: N0; Green line: N1; Dark blue line: N2; Light blue line: N3; Pink line: N4.

The value of the LAIe and PNAe derived from the NDVI exponential regression equation was used as a variable to calibrate the CERES-Rice model using the MCMC method. Table 1 shows the differences between the initialized parameters based on the remote sensing (RS)-CERES-Rice assimilation model and the original input parameters. Average differences (RE, %) between the inverted initialized parameters and the original input parameters for sowing date, seeding rate, and nitrogen amount were 1.33%, 4.75%, and 8.16%, respectively (Table 1). The RMSE values of three initial parameters between the retrieved and actual values were 1.30 d, 4.2 kg/ha, and 15.6 kg/ha based on the MCMC algorithm, respectively, after running the model 5000 times. Figure 3 shows the probability distribution of inverted initial parameters based on the MCMC method and the quantified uncertainty of the inverted initial parameters of the model.

### 2.3. Data Assimilation for LAI and PNA Estimation

**Figure 4:**
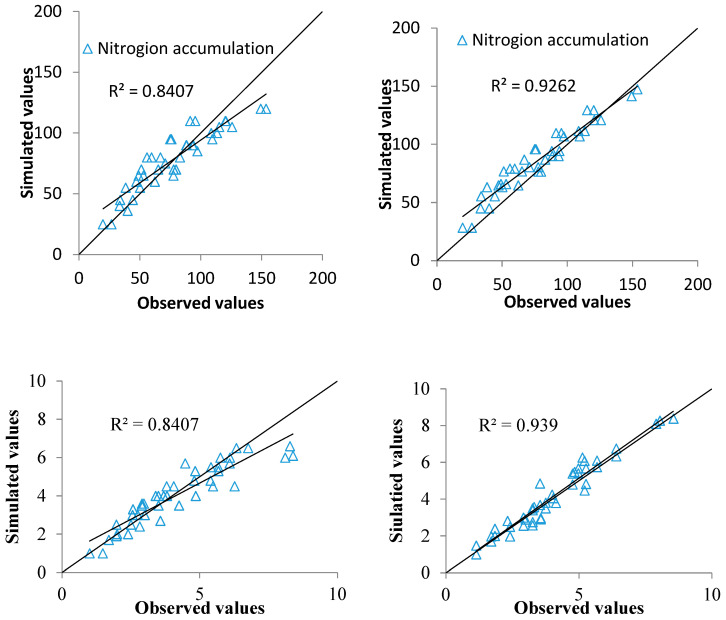
Comparisons of simulated and measured LAI and PNA values by CERES-Rice before and after assimilation based on data from experiment 3.

This technique was tested on independent datasets. The data from experiment 3 were used to validate the assimilation model. The LAI and PNA dynamics of rice were simulated by the assimilation model based on the MCMC algorithm and the optimal assimilation parameters (LAI and PNA). We compared the LAI and PNA estimated using the assimilation model with the LAI and PNA estimated using the spectral index method. The result showed that values of LAI and PNA based on the assimilation model agreed better with actual values than values simulated by the spectral index method (Figure 4). The results showed that the R^2^ values between the simulated and measured values of LAI and PNA were 0.939 and 0.926, respectively. The RMSE between the simulated and measured values of LAI and PNA were 0.74 and 7.3 kg/ha, respectively. When the GreenSeeker NDVI was used to estimate the LAI and PNA, the R^2^ values between the simulated and measured values of LAI and PNA were 0.841 and 0.84, respectively. The RMSE between the simulated and measured values of LAI and PNA were 1.32 and 13.8 kg/ha, respectively. The data assimilation model achieved better LAI and PNA estimations than the spectral index method. The result confirmed that integrating spectral indices into the DSSAT-CERES model by the MCMC data assimilation algorithm was an effective tool for PNA estimation.

### 2.4. Data Assimilation for LAI and PNA Prediction and Uncertainty Analysis

The MCMC method could quantify the uncertainty of data assimilation between crop models and remote sensing. The RMSD for LAI varies from 0.08 to 0.26, while the RMSD for PNA varies from 2.85 to 6.02. The RMSD for LAI in the tilling period is higher than in other periods, while the RMSD for LAI in the jointing period is lower than in other periods. The RMSD in the flowering period is lower than in other periods, while the RMSD for PNA in the booting period is lower than in other periods.

### 2.5. Data Assimilation for Yield Prediction and Uncertainty Analysis

**Figure 5:**
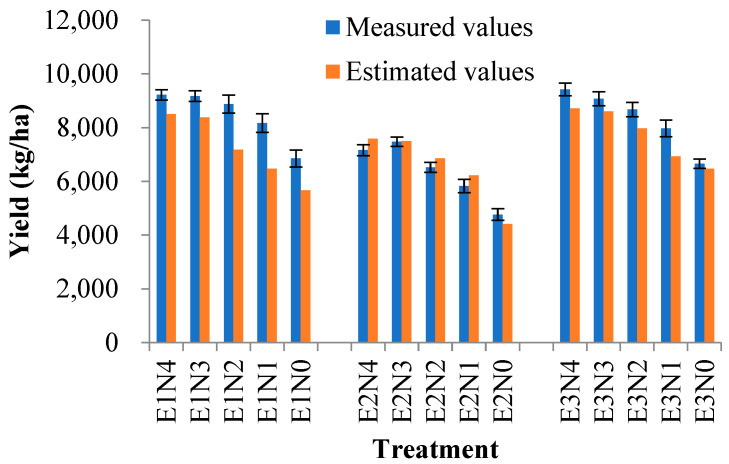
Comparisons of simulated yield by data assimilation and measured yield.

The relationship between the measured and simulated yields is shown in Figure 5. The result showed that the values of yield based on the assimilation model agreed better with the actual values. R^2^ values between the simulated and measured values of yield were 0.79. The RMSE between the simulated and measured values of yield was 661 kg/ha. The simulated yield was in agreement with the measured yield across all three experiments. The simulated yield of hybrid rice was generally underestimated. The RMSD for yield varies from 678 to 792 across all three experiments. The average RMSD for the simulated values of yield was 745 kg/ha. Yield uncertainty from data assimilation between crop models and remote sensing was quantified.

## 3. Materials and Methods

### 3.1. Experimental Design

Three experiments were conducted from 2015 to 2017 at the Zhejiang Agricultural and Forest University Modern Agricultural and Forestry Science and Technology Park in Deqing, Huzhou City, Zhejiang Province, China (120°04′ E, 30°33′ N). The field soil was classified as sandy soil and soil organic matter. The total N, available phosphate, and potassium K (0 to 25 cm soil depth) are shown in Table 2. All experiments were conducted in a randomized complete block design with three replicates for each N dressing method at a plant density of 2.55 × 10^5^ for hybrid rice and 8 × 105 plants ha^−1^ for conventional rice. Before transplanting, we applied a total of 135 kg ha^−1^ P_2_O_5_ (as Ca(H_2_PO_4_)_2_) in all experiments plus 180 kg ha^−1^ K_2_O (as KCl) to the soil in all experiments. The area of each plot was 24 m^2^ (3 m × 8 m) in all experiments.

Experiment 1: Yongyou538 was planted on 28 May 2015 with a seeding rate of 60 kg ha^−1^. Two N dressing methods were used: total N (as urea) was applied at rates of 0, 70, 140, 210, and 280 kg ha^−1^, with 50% applied at pre-planting and 50% at the jointing stages.

Experiment 2: Xiushui134 was planted on 28 May 2016 with a seeding rate of 60 kg ha^−1^. Two N dressing methods were used: total N (as urea) was applied at rates of 0, 70, 140, 210, and 280 kg ha^−1^, with 50% applied at pre-planting and 50% at the jointing stages.

Experiment 3: Yongyou1540 was planted on 30 May 2017 with a seeding rate of 60 kg ha^−1^. Two N dressing methods were used: total N (as urea) was applied at rates of 0, 70, 140, 210, and 280 kg ha^−1^, with 50% applied at pre-planting and 50% at the jointing stages.

### 3.2. Data Acquisition

#### 3.2.1. Measurement of Canopy Spectral Reflectance

A handheld GreenSeeker^TM^ (NTech Industries Inc., Ukiah, CA, USA) was used to measure canopy reflectance at the red region (656 nm) and near-infrared (NIR) region (774 nm). The NDVI was determined as NDVI = (NIR − Red)/(NIR + Red), where NIR and Red represent the fraction of emitted NIR and red radiation reflected back from the sensed area, respectively. Measurements were taken with the sensor positioned 1 m above the canopy. Readings were collected every 10 days following the jointing stages, resulting in five measurements per plot, with the average serving as a single observation.

#### 3.2.2. Plant Sampling and Analysis

After each canopy spectral reflectance measurement, five plant samples were randomly collected from each plot. The dry weight of plant organs (leaf, haulm, and grain) and the leaf area index (LAI) were measured separately, and their average values were calculated. The LAI for each plot was calculated using the specific leaf area method, which is the ratio of green leaf area to dry weight. Total nitrogen concentration in tissues was measured using the micro-Kjeldahl method [20]. Nitrogen accumulation in the above-ground parts was calculated by multiplying the above-ground dry matter (kg ha^−1^) by the above-ground plant nitrogen concentration (g kg^−1^). Grain yield for each plot was determined at maturity by harvesting 2 m^2^ of plants at a moisture content of 14%.

### 3.3. CERES-Rice Model

The Decision Support System for Agrotechnology Transfer (DSSAT 4.6) used in this research is a software tool designed for simulating crop growth and management [21]. It integrates various components such as soil, weather, and crop management practices to help agronomists and farmers make informed decisions about agricultural practices. CERES-Rice is a crop simulation model that is part of the DSSAT framework. CERES-Rice serves as a specific module within DSSAT focused on simulating rice growth and yield.

### 3.4. Integrating the MCMC Technique for Data Assimilation

**Figure 6:**
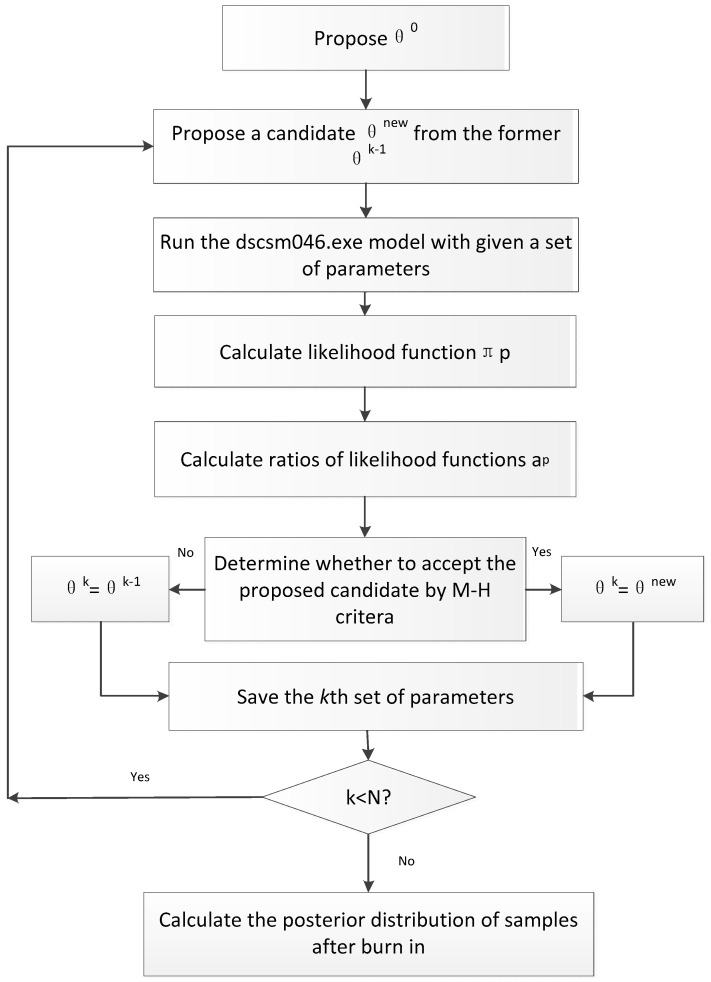
Flowchart of data assimilation based on the MCMC technique.

The MCMC technique was used to combine the CERES-Rice model and remote sensing data for rice growth estimation and yield prediction. The MCMC technique, based on the Bayesian approach, effectively synthesizes information from various sources for analyzing model uncertainties and optimizing model parameters. The Metropolis-Hastings (M-H) algorithm [22,23] is a type of MCMC technique based on Bayes’ theorem for generating samples from the posterior distribution. The principle is to generate a large enough sample from the posterior parameter distribution so that features of this distribution (expected parameter values, parameter variances) can be accurately determined. The M-H-based method for estimating region-specific cultivar parameters in this study consisted of the following steps (Figure 6).

Step 1: The adjusted parameters from this study included the sowing date, plant density, and fertilization amount. $\theta_{i}^{k}$ represented the above three parameters (i = 1, 2, 3; k = 1, 2, 3, … N). An initial set of parameters was θ^(0)^, including three initial values sampled randomly within the range of each parameter. Prior to data collection, this distribution was based on the existing knowledge about the parameter values before the measurement of new data. It was impossible to define an uninformative prior distribution if there was no available information. The probability density function of the parameters was unknown, so a uniform distribution q ($\theta_{i}^{new}$/$\theta_{i}^{k-1}$) was assumed as the prior distribution.

Step 2: Proposing a candidate of $\theta_{i}^{new}$:

(1) $$\theta_{i}^{new}=\theta_{i}^{k-1}+r\times \left(\max(\theta_{i})-\min(\theta_{i}\right))/D$$

where r was a random number uniformly distributed between 0 and 1, and max(θ_i_) and min(θ_i_) were the highest and lowest values of θ_i_, respectively. D, controlling the proposed size, was 5.

Step 3: The dscsm046.exe model was run with two sets of parameters (θ^new^ and θ^k−1^) with the required data, and the simulated LAI and PNA were calculated.

Step 4: The likelihood function π_p_(θ) was calculated with the simulated LAI and PNA values and the measured values. The likelihood function was calculated at all observation times as follows:

(2) $$\pi_{p}\left(\theta \right)\propto exp\{-\frac{1}{\sigma_{1}^{2}}\sum_{t=1}^{T}{\left[O_{1}\left(t\right)-S_{1}\left(t\right)\right]}^{2}-\frac{1}{\sigma_{2}^{2}}\sum_{t=1}^{T}{\left[O_{2}\left(t\right)-S_{2}\left(t\right)\right]}^{2}\}$$

where σ_1_ and σ_2_ were the standard deviations of actual measurements of LAI and PNA; S_1_(t) and O_1_(t) were simulated and observed LAI, respectively; S_2_(t) and O_2_(t) were simulated and observed PNA, respectively; and t stood for the different phenological stages.

Step 5: The ratio (a_p_) of the likelihood function of the above two sets of parameters was calculated as follows:

(3) $$a_{p}\left(\theta^{k-1},\theta^{new}\right)=min\{1,\frac{\left(\pi_{p}\left(\theta^{new}\right)\right)q\left(\frac{\theta^{k-1}}{\theta^{new}}\right)}{\left(\pi_{p}\left(\theta^{k-1}\right)\right)q\left(\frac{\theta^{new}}{\theta^{k-1}}\right)}\}$$

Step 6: By comparing a_p_ with a random number U [0, 1], the better candidate was chosen. If a_p_ ≥ U, set θ^k^ = θ^new^; otherwise, set θ^k^ = θ^k−1^. This was called the M-H criterion to determine whether to accept the proposed candidate.

Step 7: Steps 2–7 were repeated until k = N. N was 10,000 for a single chain.

Step 8: The samples from each chain were gathered after the burn-in (number of iterations to be discarded) of 2000 and 8000 samples were used to calculate the means and variance of the posterior distribution.

### 3.5. Statistical Analysis

Relative error (RE, %) and root mean square error (RMSE, %) were used to calculate the fitness between the simulated and measured values and evaluate the reliability of the assimilation technique. $\bar{RMSD}$ represented the dispersion among estimated values.

$$\mathrm{RE}={(O}_{j}-S_{j})/O_{j}$$

$$RMSE=\sqrt{\frac{\sum_{j=1}^{N}{\left(O_{j}-S_{j}\right)}^{2}}{N}}$$

$${RMSD}_{j}=\sqrt{\frac{\sum_{j=1}^{K}{\left(S_{j}-{\bar{S}}_{j}\right)}^{2}}{K}}$$

where O_j_ was the measured value, S_j_ was the simulated value, ${\bar{S}}_{j}$ was the average of the simulated values, and N and K were the total number of the measured values.

Microsoft Excel 2016 was used for data entry, organization, preliminary calculations, and drawing. The data were analyzed by two-way ANOVA and Duncan’s multiple range test (*p* < 0.05) using SPSS 26.0 to evaluate the dry matter, the N concentration, and the yield under different N treatments.

## 4. Discussion

### 4.1. Integration of Crop Model and Remote Sensing

The NDVI indices based on visible and red light tended to become saturated as crop stand density increased [24]. Serrano et al. [25] reported that the relationship between the NDVI and leaf area index multiplied by chlorophyll concentration (similar to N uptake) saturated at values around 1000 mg m^−2^. The normalized difference vegetation index (NDVI) became saturated for maize when LAI > 2, AGB > 3 t/ha, or PNU > 80 kg/ha [2]. Wang et al. [26] showed that the NDVI became saturated for rice when the Leaf Nitrogen Content (LNC) reached 3%. Li et al. [27] showed that canopy N accumulation was underestimated at a high LAI level. Takahashi et al. [28] showed that the spectral index displayed an obvious saturation when the plant nitrogen accumulation value of rice reached 80 kg/ha. In our research, the NDVI became saturated when the LAI reached six. The NDVI became saturated when PNA reached 90 kg/ha. The saturation effect of the NDVI was mainly due to the canopy closure, the differences in penetration into the canopy between visible light (R) and NIR, and the normalization effect embedded in the calculation formula of this index [2]. The NDVI becomes insensitive to changes in both red and NIR reflectance [3]. Therefore, the statistical model based on the relationship between remote sensing data and agronomic variables is not accurate because of the saturation of the NDVI. Some research built the relationship between the inversion agronomic variables to estimate the crop yield [29]. Crop models could continuously estimate crop LAI and Plant Nitrogen Uptake (PNU) and make up for the shortage of spectral monitoring. The data assimilation model achieved better LAI and PNA estimations than the spectral index method.

### 4.2. MCMC Method

The MCMC method is based on Monte Carlo simulations and can be directly applied to a nonlinear model [30]. Moreover, it does not rely on the Gaussian assumption of distributions as the Kalman filter-based algorithms do and is thus adapted to the potentially highly nonlinear plant/crop models [31]. The MCMC-based strategy appears to be a better choice for crop growth models in nonlinear and non-Gaussian systems [32] because it improves accuracy and efficiency and produces correct estimates of prediction uncertainty in nonlinear and non-Gaussian crop-growth model data assimilation [33]. In addition, the MCMC method can evaluate model uncertainty properly and provide a credibility interval compared to simple uncertainty analysis.

There are various specific algorithms, including the Synthetic Kalman Filter (SCE-UA) [34], Very Fast Annealing Algorithm [12], Particle Swarm Optimization Algorithm [6,15], and Ensemble Kalman Filter (EnKF) [16,17], that have been used in previous research. Too many parameters must be determined, and repeated testing is needed before the SCE-UA can be used effectively, which increases the complexity of calculations. Although annealing and particle swarm optimization are highly parallel, stochastic, and adaptive general optimization algorithms, there are some shortcomings, such as premature convergence or slow convergence in practical applications. These methods tend to fall into local optimum rather than global optimization. Moreover, none of these methods can quantify the uncertainty of data assimilation. Many studies have shown that the EnKF method has been developed for data assimilation between crop models and remote sensing data. The Kalman filter applies only to linear models with Gaussian prediction errors. Although the extended Kalman filter was developed for nonlinear dynamic models, Kalman filter-based approaches still rely on the Gaussian assumption of distribution [35]. The assimilation of the ensemble Kalman filter when processing nonlinear observation data has to simply linearize the nonlinear observations, which results in large errors and low accuracy. Chen and Cournède [31] showed that Kalman filter-based approaches suffer from the important nonlinearity of the model. 

### 4.3. Uncertainty Analysis

The MCMC method can achieve a proper evaluation of model uncertainty. The uncertainty in data assimilation not only comes from errors from the remote sensing data and model inputs of soil, weather, and field management information but also from the interaction of parameters. In the process of data assimilation, all errors can be transferred to the initial parameters of the model.

We proposed the MCMC method in a probabilistic framework in order to quantify and reduce the uncertainties in rice simulations using data assimilation techniques. The MCMC method has never been applied to complex dynamic crop models for data assimilation. The MCMC method can correct the uncertainty of data assimilation between crop models and remote sensing and then reduce errors from data assimilation algorithms [18]. This method can help us understand uncertainties in the parameters and how those uncertainties affect predictions. The uncertainty of LAI and PNA estimation is derived from the uncertainty of the initial parameters, as seen in Table 3. Table 4 shows the probability distribution of inverted initial parameters based on the MCMC method and the quantified uncertainty of the initial parameters of the model. The integration of Bayesian probability inversion and the MCMC technique is an effective method for analyzing the uncertainties in data assimilation.

### 4.4. Yield Prediction

The data assimilation method that incorporates two state variables, typically leaf area index (LAI) and plant nitrogen accumulation (PNA), demonstrated greater accuracy in estimating grain yield compared to methods using only one state variable [6,7]. The primary reason for this improvement is that the LAI plays a critical role in crop growth monitoring and yield prediction, as it reflects the extent of the crop canopy and the potential for light interception and photosynthesis. Meanwhile, the PNA serves as an important indicator of the nitrogen status in rice, which has a direct impact on photosynthetic efficiency, grain yield, and quality [7,36]. The estimated sowing date and sowing rate are slightly different from actual values and increase with rising nitrogen values. This is because the sowing date, sowing rate, and nitrogen rate are related to the LAI and PNA. There are some interactions between management parameters. We hope to improve the accuracy of model prediction and to decrease the uncertainty of model prediction. The proper number of initial parameters is important for data assimilation. Three parameters are enough to explain the variation in the LAI and PNA. Some researchers chose more initial parameters for data assimilation, but more parameters will cause larger prediction uncertainty. Too many parameters assimilated into the model will cause a co-linearity effect among parameters.

The yield of hybrid rice is higher than normal rice. Therefore, the simulated yield for hybrid rice by the CERES-Rice model is lower than the actual measured yield. The simulated yield values were overestimated at low measured yield, whereas some simulated yield values were underestimated at high measured yield. The yield reached its largest value when the nitrogen application amount was 210 kg/ha, but the yield change is not obvious with the increase in the nitrogen application rate. However, the actual yield will increase when the nitrogen application amount is more than 210 kg/ha, especially for hybrid rice. Shi et al. [37] showed that the CERES-Rice model simulated the effect of nitrogen fertilizer under a low nitrogen fertilizer application level, but it does not behave so well when excess nitrogen fertilizer is applied.

When estimating yields over large areas, genetic and cultivar parameters, water and soil characteristics, and meteorological data may bias crop growth models [38]. Farmers can use the GreenSeeker™ optical sensor to obtain canopy spectral data, which can be substituted into the calibrated model to obtain optimized parameters, further predicting the yield more accurately. Through the accurate perception of the growth status at the seedling stage and the dynamic feedback of the model, this realizes the leap from passive response to active regulation. However, this technology requires farmers to be trained, and the operation requirements are high, so the lightweight APP should be further developed to reduce the threshold for farmers and improve their acceptance.

## 5. Conclusions

This research aimed to calibrate the CERES-Rice model using LAI and PNA values derived from spectral indices, employing the MCMC technique. The initial management parameters, including sowing date, sowing rate, and nitrogen rate, were recalibrated based on the correlations between remote sensing state variables and simulated state variables. The findings indicated that the data assimilation method provided the most accurate estimations for LAI (R^2^ = 0.939 and RMSE = 0.74) and PNA (R^2^ = 0.926 and RMSE = 7.3 kg/ha) when compared to the spectral index method. Furthermore, the estimated yield closely matched the measured yield (R^2^ = 0.79 and RMSE = 876 kg/ha). Data assimilation of crop models and remote sensing data using the MCMC technique could improve the estimation of rice leaf area index (LAI), plant nitrogen accumulation (PNA), and yield by solving the saturation effect of the NDVI. The study also quantified yield uncertainty resulting from data assimilation between crop models and remote sensing. Overall, this research presents a novel approach for estimating rice growth and predicting yield by integrating the CERES-Rice model with remote sensing data through the MCMC technique. This technology can provide farmers with precise decision-making support for field management (optimizing water and fertilizer regulation, disaster early warning systems, etc.), assist governments and relevant departments in anticipating regional yield fluctuations and formulating food security strategies, and hold significant practical value for addressing climate change and stabilizing grain production.

## Figures and Tables

**Table 1:** Differences (RE, %) between the initialized parameters based on the RS-RiceGrow assimilation model and the original input parameters.

Treatments	Sowing Date (%)	Seeding Rate (%)	Nitrogen Amount (%)
E1N0	−2	−16.2	/
E1N1	−1.33	3.2	7.2
E1N2	0	17.9	−7.3
E1N3	0.67	27.2	−11.9
E1N4	1.33	32.1	1.8
E2N0	−1.6	−12.4	/
E2N1	−1.4	4.3	−5.2
E2N2	−0.7	13.6	8.4
E2N3	1	23.2	−9.3
E2N4	1.4	29.4	7.5
E3N0	−2.2	−15.2	/
E3N1	−1.3	−1	8.53
E3N2	−0.4	12.7	−3.7
E3N3	1.3	24.1	5.8
E3N4	1.2	32.5	11.7

**Table 2:** Fertilizer design and sampling date of three field trials.

Experiment No	Site	Cultivar	N Rate (Kg ha^−1^)	Planting Date	Soil Parameters
Experiment 1 2015	Deqing	Yongyou538	N0(0) N1(70) N2(140) N3(210) N4(280)	28-May	Soil organic matter: 22 g kg^−1^ Total N: 1.37 g kg^−1^ P_2_O_5_: 32.59 mg kg^−1^ K_2_O: 98.96 mg kg^−1^
Experiment 2 2016	Deqing	Xiushui134	N0(0) N1(70) N2(140) N3(210) N4(280)	28-May	Soil organic matter: 21.1 g kg^−1^ Total N: 1.27 g kg^−1^ P_2_O_5_: 38.12 mg kg^−1^ K_2_O: 90.23 mg kg^−1^
Experiment 3 2017	Deqing	Yongyou1540	N0(0) N1(70) N2(140) N3(210) N4(280)	30-May	Soil organic matter: 22.4 g kg^−1^ Total N: 1.24 g kg^−1^ P_2_O_5_: 40.14 mg kg^−1^ K_2_O: 94.56 mg kg^−1^

**Table 3:** The RMSD for the LAI and PNA.

	LAI				PNA			
	Tilling	Jointing	Booting	Flowering	Tilling	Jointing	Booting	Flowering
N0	0.17	0.10	0.16	0.15	5.30	3.28	2.92	3.07
N1	0.26	0.11	0.17	0.17	6.02	4.25	3.66	3.93
N2	0.23	0.12	0.16	0.16	5.06	3.98	3.59	3.92
N3	0.22	0.13	0.16	0.13	4.85	4.12	3.64	4.03
N4	0.15	0.13	0.13	0.14	3.84	3.85	3.61	3.78

**Table 4:** Yield prediction results by assimilated model.

	Yield		
	R^2^	RMSE (kg/ha)	RMSD (kg/ha)
E1	0.86	960	678
E2	0.83	338	764
E3	0.7	685	792
Total	0.79	661	745

## Data Availability

The original contributions presented in this study are included in the article. Further inquiries can be directed to the corresponding author.
